# Does humic acid foliar application affect growth and nutrient status of water‐stressed maize?

**DOI:** 10.1002/pei3.10156

**Published:** 2024-06-14

**Authors:** Britta Pitann, Kamran Khan, Karl H. Mühling

**Affiliations:** ^1^ Institute of Plant Nutrition and Soil Science Kiel University Kiel Germany

**Keywords:** drought, humic acids, maize, phosphorus, waterlogging, zinc

## Abstract

Maize (*Zea mays* L.) is one of the world's most important crops, but its productivity is at high risk as climate change increases the risk of water stress. Therefore, the development of mitigation strategies to combat water stress in agriculture is fundamental to ensure food security. Humic acids are known to have a positive effect on drought tolerance, but data on their efficacy under waterlogging are lacking. This study aimed to elucidate the effect of a new humic acid product, a by‐product of Ukrainian bentonite mining, on maize growth and nutrient status under waterlogging. Maize was grown for 9 weeks and three water stress treatments, which were applied for 14 days: waterlogging, alternating waterlogging and drought, and drought. On the day of stress application, the humic acid product (1% v/v) was applied to the leaves. Soil Plant Analysis Development (SPAD) values were recorded during the stress treatments. Plants were harvested after stressing ceased and fresh weight and P and Zn status were analyzed. Drought reduced shoot fresh weight, while it was unaffected under waterlogging. This is in contrast to SPAD readings, which showed a significant decrease over time under submergence, but not under drought. Under alternating stress, although SPAD values declined under waterlogging but stabilized when switched to drought, no growth reduction was apparent. Application of the humic acid product was ineffective in all cases. Although anthocyanin discoloration occurred under waterlogging stress, P deficiency, which is usually the main factor driving anthocyanin formation, was not the reason. Interestingly, Zn concentration decreased under waterlogging but not under the other stresses, which was alleviated by humic acid application. However, no effect of foliar‐applied humic acids was observed under alternating and drought stress. It can be concluded that the tested humic acid product has the potential to improve the Zn status of maize under waterlogging.

## 1 INTRODUCTION

Among all abiotic stress factors, water stress is the most important abiotic stress that suppresses crop production and thus affects global food security. Water stress in this context can be understood as either a lack of water and/or an excess of water. Due to anthropogenic climate change, an increase in extreme climate events, such as dry periods, heavy rainfall, and flash floods, is already being observed (Alifu et al., 2022; Tian et al., 2021).

Whereas in the past, water stress was considered to be predominant only in arid and semi‐arid regions of the world, it is now also observed in Germany with prolonged periods of heavy rainfall in winter, followed by pronounced periods of spring drought (Deumlich & Gericke, 2020) and thus waterlogging, whereby plants are exposed to two severe stress situations within a very short period of time.

As water is critical for plant survival, water deficits initially limit plant growth. The impact of drought on agricultural productivity thus depends on the magnitude and duration of water scarcity, as well as on plant species and developmental stages (e.g., Gupta et al., 2020; Seleiman et al., 2021 and references therein). In most cases, crops are exposed to moderate droughts caused by precipitation deficits, falling groundwater levels, and/or limited access to water supplies, leading to significant losses in overall yield, due to, for example, reduced root development, water uptake, nutrient availability and uptake, and a range of physiological changes (Ashraf, 2012; Colmer & Voesenek, 2009; Horchani et al., 2009). For example, a yield reduction of approximately 21% was observed in wheat, while the average global maize yield was reduced by almost 40% (Daryanto et al., 2016). However, also the opposite situation, that is, water surplus, is challenging crop productivity (Tabari, 2020). About 12% of the world's arable land is already affected by waterlogging, resulting in large annual economic losses (Alifu et al., 2022; Ploschuk et al., 2018). Waterlogging is accompanied by a decrease in soil oxygen level, which is the main factor causing stress and triggering a series of metabolic changes (Horchani et al., 2009). High‐yielding crops such as wheat and rapeseed are particularly susceptible to waterlogging, which can reduce yields by up to 60% for wheat and 25% for rapeseed (Hussain et al., 2023; Ploschuk et al., 2018; Wollmer et al., 2018a, 2018b).

Taken together, water stress is a serious problem in the context of food security, making it even more important to not only understand plant response mechanisms but also to identify effective strategies to mitigate water stress. Under drought conditions, these strategies include, but are not limited to, root system improvement, osmotic adjustment, and improved nutrient supply by, for example, external potassium or sulfur supply (Hussain et al., 2023; Mostofa et al., 2022; Zörb et al., 2014). Another promising approach to improve growth under drought stress is based on the study by Varanini et al. (1993), in which the use of low molecular weight humic acids resulted in the stimulation of the proton ATPase, thereby improving both growth and yield in oats. Since then, numerous studies have already shown that the addition of humic acids can improve crop growth under various abiotic stresses, such as heavy metal contamination (e.g., Song et al., 2020), salinity (e.g., Aydin et al., 2012), or drought (e.g., Chen et al., 2022). It does not matter how the humic acids are applied, directly into the soil or onto the leaf, but foliar application dominates in the majority of studies. Several hypotheses have been proposed, regardless of the type of application, to elucidate the effects of humic acids on plant growth and drought tolerance in particular. For example, a positive effect on soil chemical, physical, and biological soil properties, such as pH, soil texture and structure, soil aeration, water holding capacity, and nutrient availability, as well as microbial activity, has been attributed to humic acid application, when present in soil (Fuentes et al., 2018; Nardi et al., 2002, 2017, 2021; Shah et al., 2018; Yang et al., 2021). Humic acids may further enhance plant growth by improving root development (Jung et al., 2021) and cell membrane permeability, facilitating the uptake of essential nutrients (e.g., potassium, sulfur, nitrogen, phosphorus, and zinc) into the roots and thus the osmotic adjustment of the plant (Aydin et al., 2012; Chen et al., 2022). In addition, chlorophyll content appears to be increased after humic acid application under drought stress, maintaining photosynthesis and improving plant respiration (Nardi et al., 2002; Shen et al., 2020; Shen, Lin, et al., 2020). At the physiological level, humic acids contribute positively by increasing the synthesis of plant growth‐promoting phytohormones (e.g., auxin, cytokinin; e.g., Souza et al., 2022), activating the catalysis of plant enzymes and thus the antioxidant defense system, conferring tolerance to ROS and other co‐stressors (Billingham, 2012; Canellas et al., 2020; Laskosky et al., 2020; Nardi et al., 2002, 2021; Rose et al., 2014; van Tol de Castro et al., 2021). However, although much is already known about drought stress tolerance in combination with the use of humic acids (e.g., Jung et al., 2021) and an efficacy is irrefutable, especially with regard to soil properties when applied at high doses (Gollenbeek & van der Weide, 2020), the results in the literature are very complex. For example, there are also a number of studies in which the application of humic acids had no positive effect on plant growth (Albiach et al., 2001; Aşık et al., 2009; Bybordi & Ebrahimian, 2013; El‐Bassiouny et al., 2014; Mukherjee et al., 2014). Rather, it appears that it is the humic acid composition rather than the application rate that determines efficacy (Rose et al., 2014).

In contrast, information on the effect of humic acids under waterlogged conditions, or even a combination of waterlogging and drought stress, is scarce. However, it is known that the application of humic acids to waterlogged soils decreased the soil pH and prevented the redox potential (Eh) from further decreasing, thus reducing the bioavailability of nutrients (Fan et al., 2019).

Based on the complex results on the effect of humic acids under drought stress, as well as the lack of information on humic acids under waterlogging, this study aims to investigate the effect of a new humic acid product in mitigating water stress under different stress scenarios (drought, waterlogging, alternating stress). Maize was selected as a test crop because it is one of the most important crops worldwide in economic terms. In addition, as a dryland crop, it is particularly susceptible to waterlogging (Zaidi et al., 2003). Although breeding programs have been already initiated to induce waterlogging tolerance in maize, other non‐molecular strategies need to be considered to combat the effects of water stress on crop production. Therefore, this study aime to investigate the effect of humic acids on maize grown under drought and submergence. We hypothesized that: (1) foliar application of humic acid alleviates stress symptoms and improves plant growth not only under drought but also under waterlogged conditions; (2) foliar application of humic acids improves the nutrient status under water stress.

To the best of our knowledge, this is the first study to focus on the mitigation of waterlogging and alternating stress through the application of humic acids.

## 2 MATERIALS AND METHODS

### 2.1 Humic acid product composition

A new humic acid product was tested for its efficacy in alleviating water stress. The composition of the humic acid product (provided by AE consult) is given in Table 1.

**TABLE 1:** Humic and fulvic acids and nutrient composition of the used humic acid product.

Humic and fulvic acids			
OS	Fulvic acid/humic acid	Humic acid	Fulvic acid
(TOC %OS)		(OC %OS)	
3.52	3.52	3.43	0.9

Nutrients														
Ca	K	Mg	P_tot_	S	SO_4_^2−^	NO_3_^−^	B	Co	Zn	Mn	Cl	Na	Si	pH
(mg kg^−1^ OS)														
3630	16,300	423	458	4240	1500	<2.3	<5.0	5.4	5.3	30.6	37	154	1750	12.5

*Note*: The concentrations of heavy metals and other contaminants (e.g., polychlorinated biphenyls [PCB], polychlorinated dibenzodioxins [PCDD], and polychlorinated dibenzofurans [PCDF]) are below the critical limits.

Abbreviations: OC, organic carbon; OS, organic substance; TOC, total organic carbon.

### 2.2 Experimental design

Ten seeds of maize (*Zea mays* L. cv. Susann; Nordsaat) were sown in Mischerlich pots, filled with 6 kg of a sandy soil (Table 2). Basal fertilization was carried out as follows (g kg^−1^ soil): 1.0 NH_4_NO_3_, 1.2 KH_2_PO_4_, 1.2 KCl, 0.6 Ca(H_2_PO_4_) H_2_O, 0.33 MgSO_4_ ∙7H_2_O, 0.015 Fe‐EDTA, 0.01 CuSO_4_ 5H_2_O, 0.015 ZnSO_4_ 7H_2_O, 0.03 MnSO_4_ H_2_O, 0.01 H_3_BO_3_, 0.002 (NH_4_)_6_Mo_7_O_24_∙ 4H_2_O.

**TABLE 2:** Physico‐chemical properties of the soil.

Parameter	
Texture	Sand (Ss)
pH	5.7
Sand (%)	90.8
Silt (%)	5.9
Clay (%)	3.3
Organic matter (%)	1.7
Total nitrogen (%)	0.7
Potassium (mg 100 g^−1^)	6.7
Phosphorus (mg 100 g^−1^)	11
Sulfur (mg kg^−1^)	101.6
Zinc (mg kg^−1^)	1.7
Manganese (mg kg^−1^)	24.7
Copper (mg kg^−1^)	2.1

After germination, maize plants were isolated to three plants per pot. For proper plant development, soil moisture was maintained at 60% water holding capacity (WHC) for 6 weeks after sowing. Water stress was imposed for a total of two consecutive weeks for each of the following water stress levels: (1) control (C, 60% WHC); (2) waterlogging (WL, 100% WHC); (3) alternating waterlogging and drought stress (WL‐D 100/30% WHC30); (4) drought (D, 30% WHC). In the case of the alternating stress, a 1‐week drying phase can be assumed after the end of the WL stress during the transition from waterlogging to drought (thus allowing a total treatment duration of 3 weeks).

The humic acid product was applied on the day of stress application. For this purpose, a 1% humic acid solution (v/v) was applied abaxially and adaxially to the leaves of the maize plants at the 3‐ to 4‐leaf stage using a hand spray bottle. The soil was covered during application to prevent the spray solution from entering the soil. A wetting agent (0.1% Silwet) was added to improve adhesion of the spray solution to the leaves. Corresponding controls without humic acids were sprayed with a water–Silwet solution.

The experiment was conducted in the greenhouse under semi‐controlled conditions. The average temperature throughout the experiment was 21/16°C (day/night), relative humidity was maintained at approximately 50%–60%, and the illumination period was 16/8 h (day/night).

### 2.3 Plant sampling and analysis

Aboveground plant material was harvested immediately after stress termination and the fresh mass per pot was recorded. Each pot was treated as one biological replicate. Dry weight was determined by oven drying at 65°C to constant weight, and samples were ground to a fine powder for further analysis.

SPAD values were measured as a non‐invasive parameter of chlorophyll content at different time points using a chlorophyll meter (SPAD‐502, Konica Minolta Sensing Europe B.V.). This provides an indication of the health and N status of the plant over the experimental period.

For mineral analysis with inductively coupled plasma mass spectrometry (ICP‐MS; Agilent Technologies 7700 Series, Böblingen, Germany), 200 mg of finely ground plant material from each replicate was digested with 10 mL 69% HNO_3_ at 190°C for 45 min in a microwave oven (1800 W, MARS 6, Xpress, CEM, Matthews, MC, USA) according to the method described by Jezek et al. (2015).

### 2.4 Statistical analysis

Data were statistically analyzed using SPSS software (version 25.0). The analysis was based on five biological replicates per treatment. Treatment effects were tested using one‐way ANOVA followed by Duncan's (homogeneity of variance) or Games‐Howell (heterogeneity of variance) multiple‐range tests at *p* ≤ .05. Significant differences are indicated with different letters.

## 3 RESULTS AND DISCUSSIONS

### 3.1 Growth performance of maize under water stress and effect of humic acids after foliar application

**FIGURE 1:**
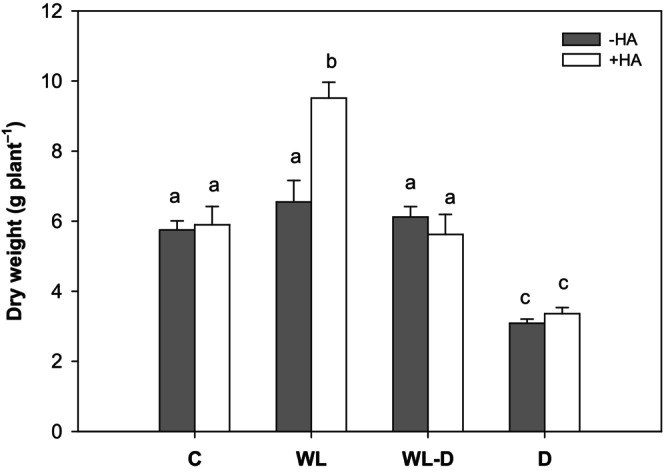
Dry weight of maize under adequate water supply (C), waterlogging (WL), alternating stress (WL‐D), and drought stress (D) conditions after foliar application of a 1% (v/v) humic acid solution. Different letters reflect significant differences (*p* < .05) between treatments (*n* = 5). +HA, with humic acid application; C, control (60% WHC); D, drought stress (30% WHC) conditions; −HA, without humic acid application; WHC, water holding capacity; WL, waterlogging stress (100% WHC); WL‐D, alternating waterlogging‐drought stress (100/30% WHC).

**FIGURE 2:**
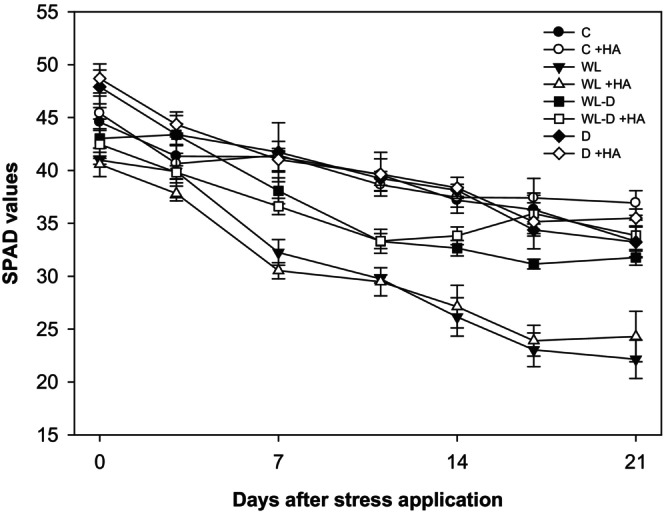
Effect of HA humic acid foliar application (1%, v/v) on SPAD values of maize under adequate water supply (C), waterlogging (WL), alternating stress (WL‐D), and drought stress (D) conditions. +HA, with humic acid application; C, control (60% WHC); D, drought stress (30% WHC) conditions; −HA, without humic acid application; WHC, water holding capacity; WL, waterlogging stress (100% WHC); WL‐D, alternating waterlogging‐drought stress (100/30% WHC).

Water stress, particularly water deficit, is the abiotic stressor that has the greatest impact on plant growth. Despite being a dryland crop, maize is also highly susceptible to drought, as evidenced by numerous studies (e.g., Daryanto et al., 2016; Kamara et al., 2003; Khalili et al., 2013). This was corroborated in the present study, wherein maize growth was diminished by approximately 50% in comparison to the corresponding control (Figure 1). Despite maize's requirement for substantial quantities of water, it is not tolerant of submergence when soil moisture exceeds 80% (Chen et al., 1988). It was therefore anticipated that the effect on plant growth observed in drought conditions would also be evident in waterlogging conditions, as has been described for various sensitive crops (Araki et al., 2012; Hussain et al., 2022, 2023; Ploschuk et al., 2018; Setter & Waters, 2003; Wollmer et al., 2018a, 2018b). However, no such effect was observed in this study. Both waterlogging and alternating stress (waterlogging + drought) resulted in similar fresh weights as the control (Figure 1). This is surprising, as other studies on maize showed that even only 3–6 days of exposure to excess water significantly limited growth (Ren et al., 2014). This finding is consistent with other studies on maize (Huang et al., 2022; Przywara & Stępniewski, 1999; Yang & Chen, 1998), which demonstrated that prolonged waterlogging (12 and 15 days, respectively, comparable to the duration of this experiment) resulted in growth depression. One potential explanation for the observed growth depression is a reduction in photosynthetic activity (Tian et al., 2019). Waterlogging‐induced anoxia/hypoxia in the roots leads to the formation of reactive oxygen species (ROS), which causes membrane oxidation and chloroplast degradation (Hasanuzzaman et al., 2017; Ren et al., 2016). However, this effect can be excluded as, although SPAD values showed the most pronounced decline during the sole waterlogging treatment and in the initial phase of alternate stress (Figure 2), there was no correlation with growth (Figure 1).

Although growth was not affected by waterlogging or alternating stress, it remains unclear whether foliar humic acid application is beneficial, given the well‐documented alleviating effects of humic acids on crop growth. Humic acids are classified as important biostimulants, which improve soil fertility at the biological, physical, and chemical levels (Bakry et al., 2009; Shah et al., 2018). Additionally, they enhance numerous processes in plants, including photosynthesis, respiration, permeability of cell membranes, and nutrient uptake (Nardi et al., 2002; Zhang et al., 2014). Unfortunately, no such positive effect of foliar application of humic acids on maize growth or SPAD values could be observed, especially under drought stress, but also alternate stress (Figure 1), as previously described by Kamyab et al. (2016) for turf grasses. This is surprising, given that many examples can be found in the current literature where foliar application of humic acids has led to significant improvements in the growth of various crops. These include canola (Lotfi et al., 2015, 2018), maize (Moghadam et al., 2014), potato (Alenazi et al., 2016), and sugar beet (Khodadadi et al., 2020). Nevertheless, there is a positive effect on growth under waterlogging (WL; Figure 1), despite the initial lack of impact of waterlogging on growth.

### 3.2 Effect of humic acids on nutrient status under water stress

**FIGURE 3:**
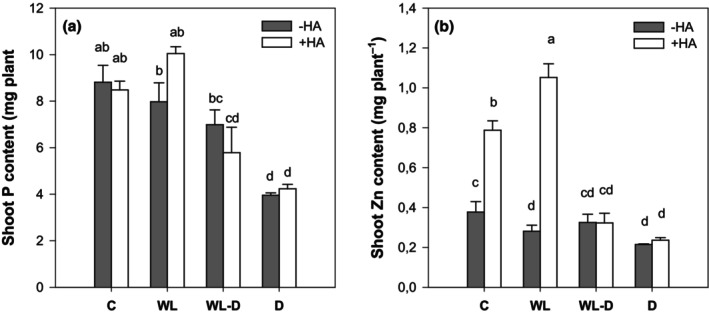
Effect of humic acid foliar application (1%, v/v) on shoot P (a) and Zn (b) concentration under adequate water supply (C), waterlogging (WL), alternating stress (WL‐D), and drought stress (D) conditions. Different letters reflect significant differences (*p* < .05) between treatments (*n* = 5). +HA, with humic acid application; C, control (60% WHC); D, drought stress (30% WHC) conditions; −HA, without humic acid application; WHC, water holding capacity; WL, waterlogging stress (100% WHC); WL‐D, alternating waterlogging‐drought stress (100/30% WHC).

While drought is defined by a lack of water and thus higher oxygen levels in soil, in waterlogged soils gaseous exchange and gas diffusion are impeded (Jackson & Drew, 1984; Striker, 2012). As a consequence of hypoxic/anoxic soil conditions, stomatal conductance is affected, which in turn impedes water and nutrient uptake and translocation are hampered (Colmer & Voesenek, 2009; Jackson & Drew, 1984). This effect is further compounded by a decline in the soil redox potential, which also affects nutrient transformation and availability, including phosphorus (P) and micronutrients (Patrick & Mahapatra, 1968; Wollmer et al., 2019). As ferric phosphates are reduced under O_2_–depleted conditions, phosphorus desorbs and soil water concentrations markedly increase (Patrick & Mahapatra, 1968). However, in the context of alternating stress, the drying of a soil subsequent to submergence has been observed to result in a decrease in P solubility. This phenomenon may be attributed to the re‐oxidation of iron (Fe), which in turn increases the P‐fixing capacity (He & Dijkstra, 2014; Patrick & Mahapatra, 1968 and references therein). This is consistent with the findings of the present study, which demonstrated a significant decrease in P concentration under waterlogging (data not shown). It is noteworthy that, in the sole waterlogging treatment, the anticipated increase in P concentration was not observed, but rather a slight decline. However, this must be attributed to a dilution effect, as plant biomass was high, finally resulting in a P content comparable to the control (Figure 3a). In contrast, drought conditions result in a reduction in P availability, which is accompanied by a decrease in uptake. However, when corrected for plant biomass, the P content is significantly reduced in comparison with the well‐watered control (Figure 3a). For all treatments, no significant effect of humic acids on P concentration was observed (Figure 3a).

**FIGURE 4:**
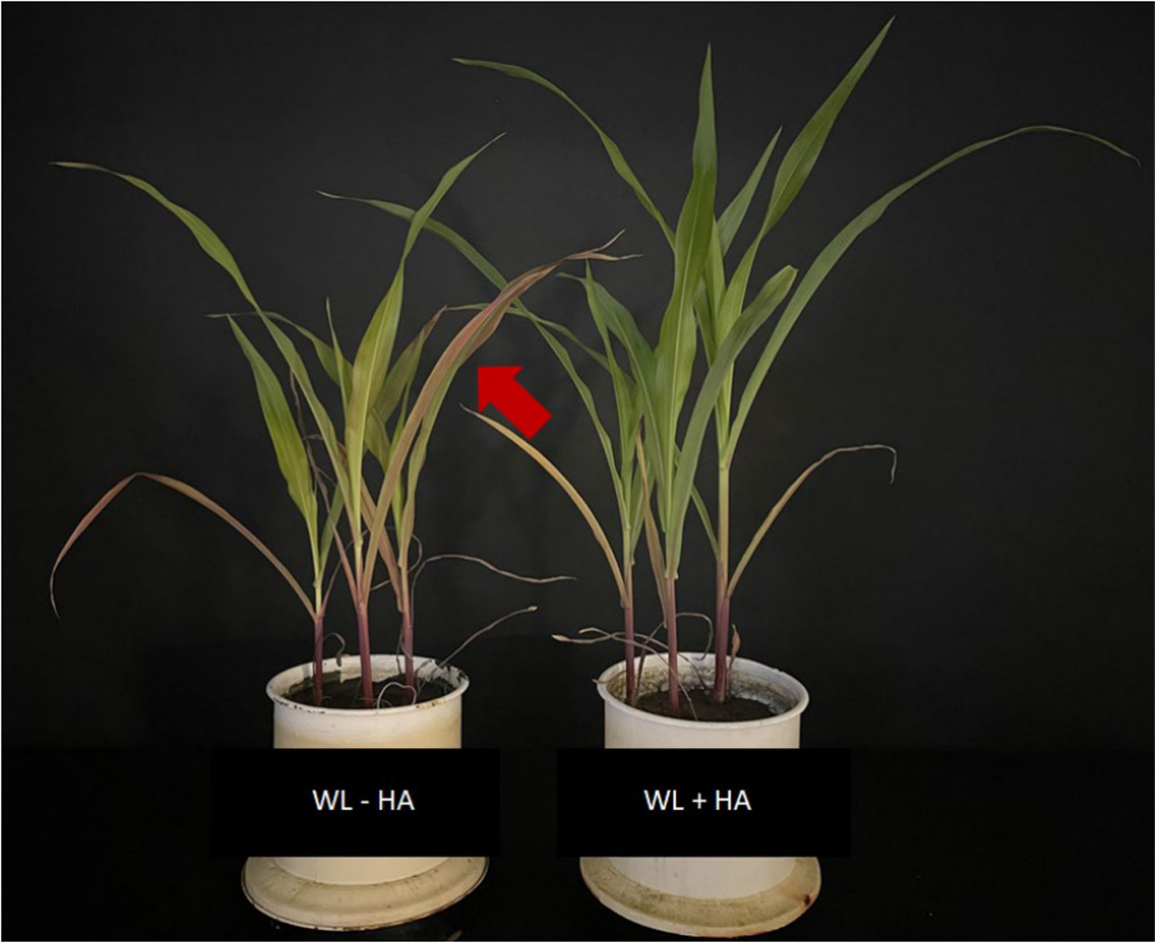
Effect of humic acid foliar application (1%, v/v) on anthocyanin discoloration of maize under waterlogging (WL) conditions. The arrow indicates anthocyanin discoloration of maize leaves. +HA, with humic acid application; −HA, without humic acid application; WL, waterlogging (100% WHC).

Interestingly, waterlogged plants exhibited a distinct anthocyanin discoloration (Figure 4), a symptom typically associated with P deficiency. However, the P content in maize under submergence does not indicate any P deficiency that could explain anthocyanin formation. For instance, Sun et al. (2016) demonstrated that distinct maize hybrids exhibited disparate anthocyanin concentrations, which were solely produced when the P content declined below a critical threshold value in QXH0121, whereas QXN233 was not responsive to low P. In conjunction with the non‐differential P content to the control (Figure 3a), we conclude that P deficiency did not occur, yet we raise the question of why anthocyanins were formed? To address this, it is necessary to consider that the discoloration of anthocyanins was reduced following the application of humic acids (Figure 4).

Anthocyanin formation is a common strategy employed by plants to combat various (a)biotic stresses, including oxidative stress and nutrient deficiencies (Chalker‐Scott, 1999; Ding et al., 2022; Gould, 2004; Kobayashi et al., 2013; Lawanson et al., 1972; Løvdal et al., 2010). The formation of reactive oxygen species (ROS) under water stress and its effect on the photosynthetic apparatus have been well documented in the literature (Hasanuzzaman et al., 2017; Ren et al., 2016). This is consistent with the observed decline in SPAD values under submergence and the initial phase of alternating stress (Figure 2), which may partially be attributed to photooxidation of chloroplasts. Ding et al. (2022) recently demonstrated that in an *anthocyanin‐more* (*am*) mutant of rapeseed (*Brassica napus*), tolerance against waterlogging was associated with higher anthocyanin levels. It can be postulated that anthocyanin formation represents a primary response in waterlogged maize, serving to combat oxidative stress.

The effect of anthocyanin formation may also be associated with the reduction of Zn in the shoot under conditions of waterlogging (Figure 3b). Zinc is a metal that exhibits relatively high mobility in soil, due to the formation of outer‐sphere weak bonds (Marilú Barrera et al., 2021). Consequently, as the redox potential of the soil declines under submergence, the concentration of Zn in pore water and leachate increases, due to a higher desorption rate (Charlatchka & Cambier, 2000; Du Laing et al., 2007; Olivie‐Lauquet et al., 2001). Consequently, the reduction in shoot Zn concentration, observed here, provides evidence that Zn is being mobilized and leached out, rendering it unavailable to the plant. Zinc plays a fundamental role in plants, particularly under abiotic stress, as it is involved in a number of physiological and molecular processes (e.g., Umair Hassan et al., 2020 and references therein). For example, Zn plays a pivotal role in the antioxidative defense system of plants, which protects them from oxidative stress by activating, for example, superoxide dismutase (SOD). Furthermore, anthocyanins are known to be associated with the plant Zn status. As Zn levels increase, anthocyanin formation decreases and vice versa (Asad et al., 2014; Kösesakal et al., 2009). It can be reasonably inferred that Zn deficiency was the underlying cause of the observed anthocyanin discoloration in waterlogged conditions, as it compensated for the detoxification of ROS by lacking Zn. Conversely, the Zn concentration of the humic acid product (Table 2) indicates that humic acid leaf application led to a markedly increased Zn concentration in control and waterlogging treatments (Figure 3b). Consequently, anthocyanin discoloration disappeared in WL, which may be attributed to Zn being sufficiently resupplied under waterlogging. However, when waterlogging is ceased, as reflected by alternate stress in this study, Zn mobilization is reverted (Charlatchka & Cambier, 2000; Du Laing et al., 2007), shifting the redox potential towards more oxidizing conditions and binding to, for example, sulfates in soil (Marilú Barrera et al., 2021). This may then prevent further leaching of Zn, which would explain the Zn concentration being at the same level as the control treatment (Figure 3b).

However, in this study, a similar and also well described positive effect of humic acid application on plant growth under drought (e.g., Chen et al., 2022; Khodadadi et al., 2020; Lotfi et al., 2015; Moghadam et al., 2014) could not be shown. The observed discrepancies in humic acid effectiveness under drought conditions can be attributed to the fact that the majority of studies employed (almost) unfertilized soil or growth substrates, with no basal fertilization or only NPK being applied to the respective growth substrates. In contrast, the soil used in this study was optimally supplied with all relevant macro‐ and micronutrients (see Section 2.2). Moreover, the application concentrations of up to 30% humic acids (w/v) were utilized in numerous studies, in contrast to the comparatively low concentration of 1% humic acids (v/v) used in the present study. Although effective under waterlogging conditions, it appears insufficient to be effective under drought. Furthermore, most studies lack information regarding the prevention of humic acids leaking from leaves into soil. This is evidenced by the lack of information regarding the use of shielding the soil during spraying or the use of a wetting agent as in this study, to increase adhesion. It must therefore be assumed that the favorable outcomes observed in other studies under drought conditions are likely to be the result of a fertilization effect, which has compensated for any nutrient deficiencies that may have existed from the outset of the experiments.

## 4 CONCLUSIONS

To date, the consensus has been that the application of humic acids has a positive effect under drought stress. However, based on the results of this study, foliar application of at least the humic acid product used here does not seem promising, with no effect on SPAD values and nutrient status.

However, for the first time, a positive effect of humic acids on plant growth was shown under waterlogging stress. Anthocyanin discoloration disappeared after foliar application of the humic acid product, coinciding with an increase in Zn status, which was otherwise significantly reduced under waterlogged conditions, suggesting a direct relationship between waterlogging and the role of Zn in plant metabolism.

Therefore, based on this initial study, we postulate that foliar application of humic acids, depending on its composition and application rate, has the potential to mitigate waterlogging stress and thus maintain plant productivity under temporary submergence.

## FUNDING INFORMATION

None.

## CONFLICT OF INTEREST STATEMENT

The authors have no potential conflict of interest to declare.

## Data Availability

The data that support the findings of this study are available from the corresponding author upon reasonable request.
